# Pulsed Electromagnetic Field Alleviates Intervertebral Disc Degeneration by Activating Sirt1-Autophagy Signaling Network

**DOI:** 10.3389/fbioe.2022.853872

**Published:** 2022-03-21

**Authors:** Yi Zheng, Liangwei Mei, Shengyou Li, Teng Ma, Bing Xia, Yiming Hao, Xue Gao, Bin Wei, Yitao Wei, Da Jing, Zhuojing Luo, Jinghui Huang

**Affiliations:** ^1^ Department of Orthopedics, Xijing Hospital, Fourth Military Medical University, Xi’an, China; ^2^ Faculty of Life Sciences, Northwest University, Xi’an, China; ^3^ Department of Biomedical Engineering, Fourth Military Medical University, Xi’an, China

**Keywords:** pulsed electromagnetic fields, intervertebral disc degeneration, autophagy, SIRT1, extracellular matrix (ECM)

## Abstract

Intervertebral disc (IVD) degeneration is regarded as a major contributor to low back pain (LBP), causing serious economic burden on individuals and society. Unfortunately, there are limited effective treatment for IVD degeneration. Pulsed electromagnetic field (PEMF) is an economical and effective physical therapy method, with reduced side-effects. It offers certain protection to a number of degenerative diseases. Therefore, understanding the underlying mechanism of PEMF on IVD is important for improving the PEMF therapeutic efficiency. In this study, PEMF up-regulated extracellular matrix (ECM) related genes in degenerated nucleus pulposus (NP) cells. It also increased SIRT1 expression and promoted autophagy in degenerated NP cells. In contrast, the autophagy suppressor 3-methyladenine (3-MA) reversed the beneficial effect of PEMF on ECM production. Similarly, the SIRT1 enzyme activity suppressor EX 527 also inhibited the effect of PEMF on autophagy and ECM production in NP cells, thereby suggesting that PEMF regulated ECM related genes expression through SIRT1-autophagy signaling pathway. Lastly, PEMF significantly reduced IVD degeneration in a rat model of IVD degeneration *in vivo*. In summary, our study uncovers a critical role of SIRT1-dependent autophagy signaling pathway in ECM protection and thus in the establishment of therapeutic effect of PEMF on IVD degeneration.

## Introduction

Low back pain (LBP) is an age-related condition, the prevalence and impact of which are increasing year by year (Cashin et al., 2021; Knezevic et al., 2021). In a recent global survey involving 50 chronic diseases, LBP ranked the first cause of disability in the elderly (Cieza et al., 2021). Although LBP has multifactorial etiology, intervertebral disc (IVD) degeneration is believed to be the main contributor of LBP pathology (Foster et al., 2018; Liu et al., 2021). IVD consists of three distinct regions, namely, the central nucleus pulposus (NP), which is a gel-like tissue rich in aggrecan; the annulus fibrosus (AF), which is mainly composed of collagen fibers; and the cartilage endplate (CEP), which is connected to vertebral bodies. Existing studies revealed that alterations in extracellular matrix (ECM), inflammation, enhanced catabolism, and cellular aging and death promote IVD degeneration (Tu et al., 2021; Francisco et al., 2022). Despite global prevalence of IVD degeneration, there are no effective treatments available for IVD degeneration and its related pathologies.

Pulsed electromagnetic field (PEMF), as an economical and non-invasive form of physical therapy, with reduced side-effects. It has been used in clinical practice since the 1970s, mainly for the treatment of delayed fracture healing, nonunion, and osteonecrosis (Caliogna et al., 2021; Varani et al., 2021). Numerous studies confirmed that PEMF possesses anti-inflammatory and anti-aging properties, and it promotes ECM synthesis both *in vitro* and *in vivo* (Kavand et al., 2016; Zhi et al., 2016; Parate et al., 2017; Bloise et al., 2020). Recent evidences revealed that PEMF serves a certain function in inhibiting IVD degeneration in a short-term treatment. Miller et al., for instance, reported that PEMF lowers levels of inflammatory factors and catabolites, while enhancing anabolism *in vitro* (Miller et al., 2016). In addition, Chan, AK et al. validated that PEMF suppresses levels of inflammatory factors and accelerates ECM production in a rat tail-puncture induced IVD degeneration model in a 7-day treatment (Chan et al., 2019). Although existing studies demonstrated certain clinical benefits of PEMF, the mechanism whereby PEMF regulates IVD degeneration remains unknown.

Interestingly, recent studies have uncovered that PEMF show a potential role in regulating silent information regulator 1 (SIRT1), a NAD + -dependent deacetylase, which might activate autophagy in several tissues (Jeong et al., 2017; Patruno et al., 2020). Autophagy serves an essential function in cell homeostasis by removing non-functional cellular components and organelles. During aging process, autophagy related genes dramatically decrease in IVD tissues (Kritschil et al., 2021). Prior studies have revealed that autophagy protects against IVD degeneration (Hu J. et al., 2021; Dai et al., 2021; Zhong et al., 2021). Hence, activating autophagy may be an effective treatment against IVD degeneration. Emerging evidences have confirmed that PEMF exerts therapeutic effects by activating autophagy (Jiang et al., 2016; Zielinski et al., 2020). However, the role of autophagy in the beneficial effect of PEMF on IVD degeneration is currently unknown, and underlying mechanisms have not yet been fully clarified. Therefore, the present study aimed to evaluate whether PEMF alleviate IVD degeneration *via* SIRT1-mediated autophagy in cellular and animal models.

## Materials and Method

### PEMF Stimulators

**FIGURE 1:**
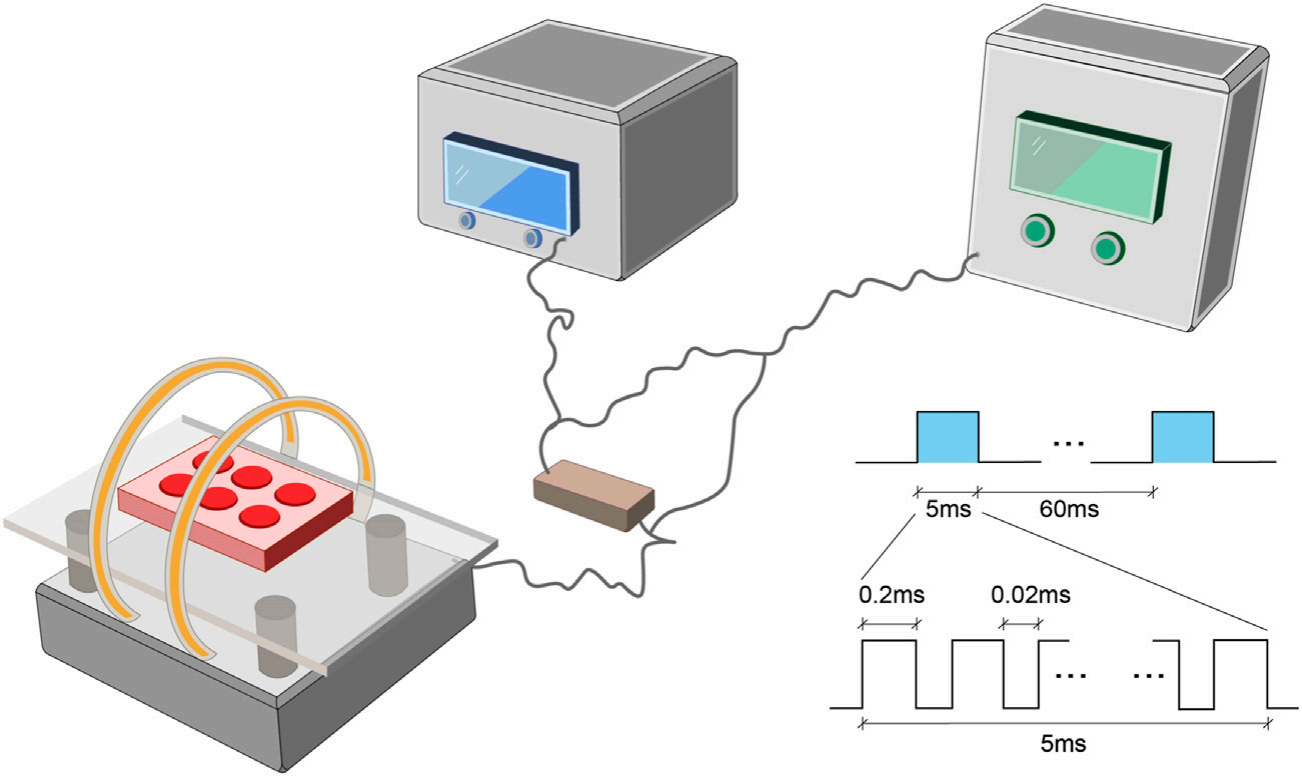
The PEMF exposure system and output waveform. The PEMF device was composed of a signal generator with two Helmholtz coils. A compact 2 Ω resistor was carefully positioned in series with coils. The PEMF waveform was composed of a pulsed burst (burst width, 5 ms; pulse width, 0.2 ms; pulse wait, 0.02 ms; burst wait, 60 ms; pulse rise, 0.3 µs; pulse fall, 2.0 µs), frequented at 15 Hz. PEMF, Pulsed Electromagnetic Fields.

The PEMF stimulation system (GHY-III, Airforce Military Medical University (AMMU), Xi’an, China; China Patent no. ZL02224739.4) consisted of a PEMF generator and Helmholtz coils (Figure 1). In cellular experiments, we employed Helmholtz coils with 200 mm diameters and an interval distance of 100 mm. Each coil was composed of 0.8 mm diameter enameled coated copper wire. The PEMF waveform comprised of a pulsed burst (burst width 5 ms; pulse width, 0.2 ms; pulse wait, 0.02 ms; burst wait, 60 ms; pulse rise and fall times: 0.3 µs, 2.0 µs), which was frequented at 15 Hz. This waveform is known to exert a therapeutic effect on nerve injury and osteoporosis, as is evidenced in our prior investigations (Jing et al., 2010; Zhu et al., 2017). A compact 2 Ω resistor was introduced in series connection, accompanying coils. The wave shape and frequency were assessed with an oscilloscope (6,000 series; Agilent Technologies, United States), and a Gaussmeter (Model 455 DSP; Lake Shore Cryotronics) was employed to monitor the precision of PEMF output. NP cells received PEMF stimulation for 4 h/day. In animal experiments, different Helmholtz coils were used, carrying an interval distance of 304, and diameter of 800 mm but the PEMF output was the same in *in vitro* and *in vivo* studies, which was confirmed by the oscilloscope and Gaussmeter test.

### NP Cells Isolation, Culture, and Treatment

Our work received ethical approval from the Xijing Hospital, and we received informed consent from all eligible participants. Human intervertebral disc tissue was obtained from patients with lumbar disc herniation (n = 8, age 59.0 ± 0.5 years, range 55–74 years) and fresh NP tissue samples were obtained during the surgery. Samples were washed thrice with normal saline, and cut into 2 mm^3^, before digestion for 30 min in 0.2% pancreatin. They were then rinsed thrice in PBS and incubated in 0.25% type II collagenase for 4 h, with shaking every half an hour, before terminating digestion. Any undigested tissue was then filtered with a 70-micron filter, before resuspending the digested tissue in DMEM/F12 medium with 10% fetal bovine serum and antibiotics, prior to culture in a 5% CO_2_ humidified incubator at 37°C. Cells received PEMF treatment 4 h per day, and samples were collected at 0, 24, 48, and 96 h to test apoptosis rate. In order to examine PEMF-mediated autophagy *via* SIRT1, cells were pretreated with either 10 mM 3-MA (an autophagy suppressor) or EX-527 (a SIRT1 enzymatic activity suppressor) for 2 h before the first PEMF stimulation and then maintained in the same media for the entire experiment. All experiments were repeated thrice for good measure.

### Cell Viability Assay

NP cells were seeded to the 96-well plate (density: 5 × 10^4^/ml). In 24, 48, and 96 h timepoint, each well was washed with PBS and then treated using 10-µl of Cell Counting Kit 8 (CCK8; Dojindo, Kumamoto, Japan) for1 h at 37°C. The optical density was then evaluated *via* a microplate reader (BioTek, United States) at 450 nm absorbance.

### RNA Extraction and Quantitative Real-Time Polymerase Chain Reaction (qRT-PCR)

Total RNA was extracted from NP cells using the Total RNA Mini Kit (QIAGEN, Germany), following kit directions, then converted to cDNA, prior to qPCR analysis *via* SYBR Green PCR Master Mix (TAKARA, Japan) and a BioRad CFX96 PCR System (BioRad, Australia). GAPDH served as an endogenous control. The primers used for qRT-PCR reactions were as follows: collagen II (F)5′-CGAGGCAGACAGTACCTTGA-3′, (R) 5′-TGC​TCT​CGA​TCT​GGT​TGT​TC-3’; MMP3 (F) 5′-GCT​CAT​CCT​ACC​CAT​TGC​AT-3′, (R) 5′-GCT​TCC​CTG​TCA​TCT​TCA​GC-3’; GAPDH (F) 5′-GGCACAGTCAAGGCTFAGAATG-3′, (R) 5′-GGT​GGT​GAA​GAC​GCC​AGT​A-3’.

### Western Blot Assay

NP cells were thrice rinsed in PBS, before harvest using RIPA buffer and centrifugation at 12,000 g for 15 min. Protein quantification was done with a BCA analysis kit. Proteins were electrophoresized *via* SDS-PAGE, before transfer to PVDF membranes (Thermo Fisher Scientific). Next, they were blocked using 5% non-fat milk in TBS-Tween (TBST) at room temperature (RT) for 1 h, before exposure to primary antibodies (namely, p62, SIRT1, LC3B, collagen II, MMP-3, and GAPDH (all from abcam and has a dilution of 1:1,000)) at 4°C overnight (O/N). They were next exposed to secondary HRP-linked antibodies, either goat anti-rabbit or goat anti-mouse secondary antibodies (Cwbiotech, Beijing, China) at a 1:2,000 dilution for 1 h, before protein band detection using chemiluminescence (Bio-Rad).

### Immunofluorescence Staining

NP cells underwent fixation in 4% paraformaldehyde for 10–15 min, and permeabilization in 0.2% Triton X-100 for 20 min, before blocking in 10% normal goat serum at RT for 1h, and O/N incubation in antibodies against collagen II (1:200) and MMP-3 (1:150) at 4°C. Then, they were thrice washed in PBS before exposure to secondary antibodies (FITC or Cy-3, 1:200) for 1 h at RT, and to DAPI for 10 min. Cells underwent three PBS washes for 15 min after every step. Lastly, they were visualized under a fluorescence microscope and analyzed using the ImageJ software.

### Transmission Electron Microscopy

A TEM (H-7650; Hitachi, Tokyo, Japan) was employed for calculating autophagic vesicles within NP cells. In short, NP cells underwent O/N fixation in 2.5% glutaraldehyde, followed by a 1 h post-fixation in 2% OsO_4_, before receiving gradient dehydration in acetone, followed by embedding, and slicing into 70 nm ultrathin sections using LKB-V ultrathin microtome. Subsequently, they were post-stained with uranyl acetate and lead citrate, and visualized with TEM (H-7650; Hitachi, Tokyo, Japan).

### Surgical Procedure

All animal experiments abided by the Institutional Ethical Committee of Fourth Military Medical University guidelines. Fifteen rats were arbitrarily separated into three groups: control (n = 5); needle puncture (n = 5); and needle puncture and PEMF treatment (n = 5). All rats were intraperitoneally anesthetized with pentobarbital solution (1%, 40 mg/kg). As described in previous studies, following tail skin disinfection with iodophor and alcohol, a 20-gauge needle was used to fully puncture the tail disc from Co6-Co8 levels, including two IVDs (Co6-Co7 and Co7-Co8) (Sun et al., 2020; Mei et al., 2021). The needle was then rotated 180 and left for 1 min before removal. Following the operation, all the animals were placed back in their cages and provided with ample food and water. The rats in the experimental group were provided with PEMF treatment (8:00–12:00, 4 h/d) immediately after surgery. Rats were housed in a climate-controlled facility (24 ± 1°C, 35–60% humidity; 12 h light/dark cycle).

### Magnetic Resonance Imaging

MRI images were captured 8 weeks after treatment, using an MRI system (Siemens 3 T Magnetom Trio Tim scanner, Munich, Germany). The IVD degeneration score was computed by two independent spinal surgeons, based on the Pfirrmann grading system, ranging from grade I to grade V.

### Histopathologic Analysis

Upon fixation in 4% paraformaldehyde, the IVD tissue was decalcified, paraffin-embedded, and sliced into 7 μm thick sagittal sections, then stained with Hematoxylin and Eosin (H&E), Safranin O-Fast Green (SOFG), and Sirius Red. The histological scores, including NP morphology and cellularity, AF organization, matrix structure and CEP integrity, were determined by independent researchers who were blinded to the study, as reported previously (Sobajima et al., 2005; Chan et al., 2019).

### Statistical Analysis

All data were analyzed using one-way ANOVA from the SPSS 13.0 software (IBM, IL, United States) and expressed as means ± standard deviations (SDs). Tukey’s post hoc and Dunnett’s tests were employed for significance comparisons (GraphPad Prism 7.0; GraphPad Software, CA, United States). *p* < 0.05 was deemed significant.

## Results

### The Effect of PEMF on the Characterizations and Anti-degeneration of NP Cells

**FIGURE 2:**
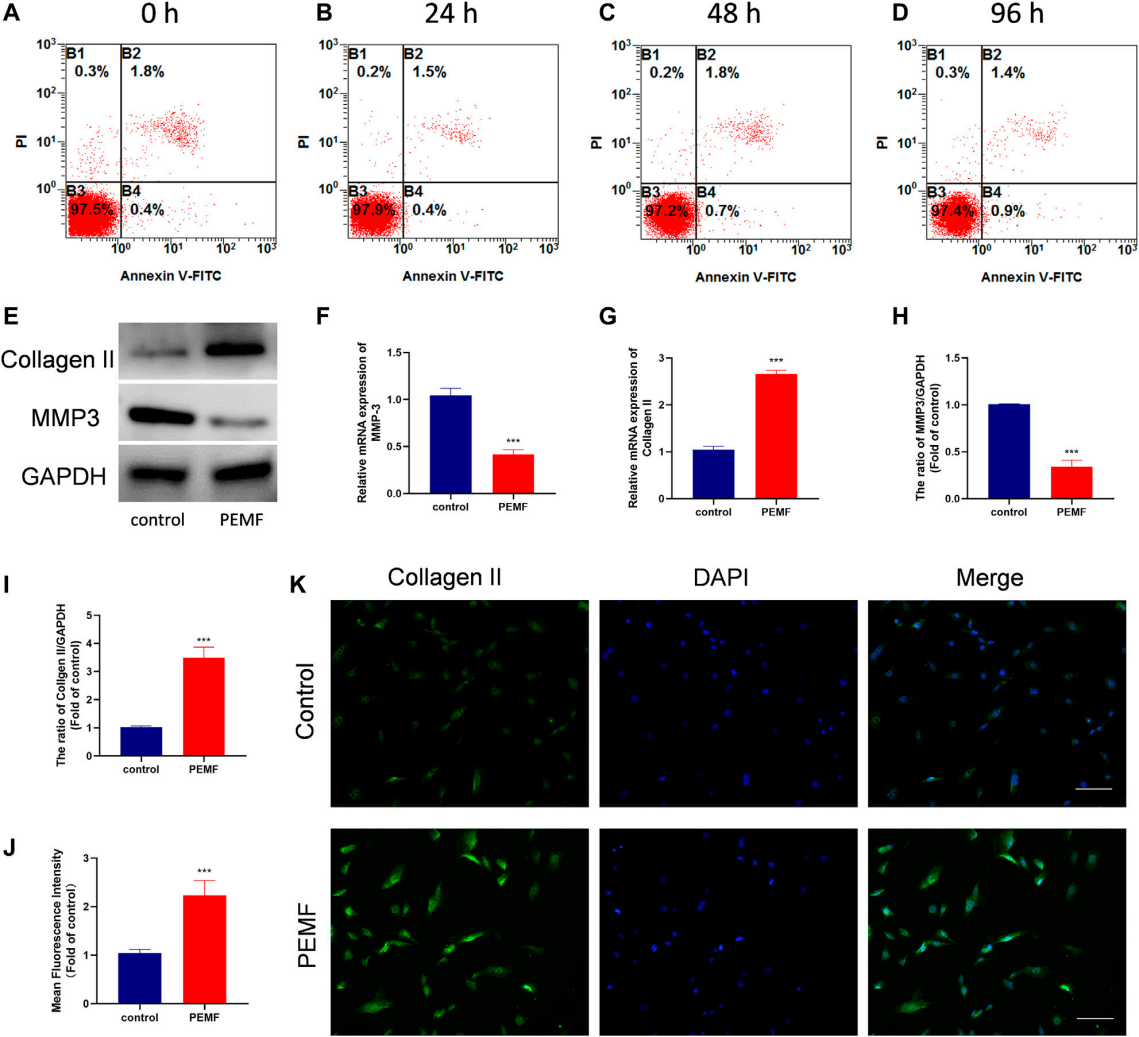
PEMF attenuates cell degeneration in human degenerated NP cells. **(A–D)** The apoptosis ratio of NP cells at time points 24, 48, and 96 h after 4 h/day of PEMF administration. **(E–I)** The qRT-PCR analysis of Collagen II and MMP-3 transcripts (n = 3), western blot and semi-quantitative analyses of Collagen II and MMP-3 protein levels (n = 3). **(J–K)** Representative images of immunofluorescence and semi-quantitative analyses of Collagen II fluorescence intensity in NP cells, imaged by fluorescence microscopy (scale bar = 50 μm). ***p < 0.001. Data presented as means ± SD.

To examine the PEMF-mediated effect on NP cell apoptosis, viability and cell morphology, we stimulated NP cells with PEMF 4 h/day for four consecutive days, and monitored cell apoptosis and viability using flow cytometry and CCK-8 assay at 0, 24, 48, and 96 h. Based on our results, PEMF did not exert any obvious cytotoxicity on NP cells (Figures 2A-D and Supplementary Figure 1A). Moreover, phase-contrast microscopy results showed that PEMF didn’t change cell morphology at 96 h timepoint (Supplementary Figure 1B and 1C).

We also assessed the PEMF-mediated regulation of NP cell ECM related genes expression. Following 4 days of treatment, RT-qPCR data revealed that PEMF markedly enhanced collagen II expression, while decreasing Mmp3 levels in NP cells (Figures 2F,G). Results from western blot analysis further corroborated the RT-qPCR data (Figures 2E,H,I). In addition, using immunofluorescence staining and semi-quantitative analysis, we also revealed that PEMF markedly elevated collagen II expression in NP cells (Figures 2J,K). Collectively, these data suggest that PEMF delays the IVD degeneration process *in vitro*.

### PEMF Activates Autophagy and Elevates SIRT1 Levels in Degenerated NP Cells

**FIGURE 3:**
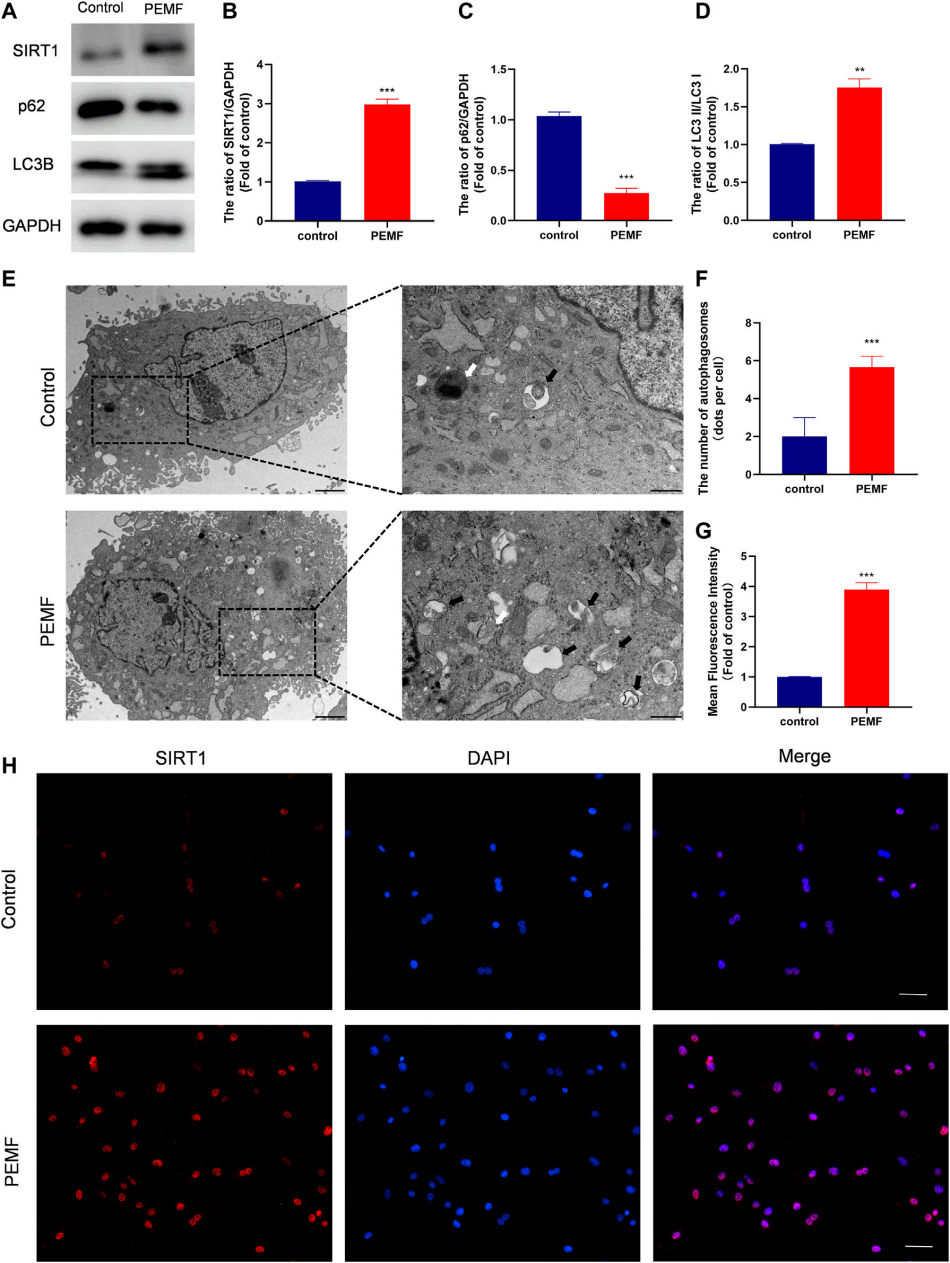
PEMF exposure promotes autophagy and elevates SIRT1 levels and activity in the human degenerated NP cells. **(A–D)** Western blot and semi-quantitative analyses of SIRT1, p62, and LC3B protein levels (n = 3). **(E–F)** Autophagosomes and autophagolysosomes, visualized by transmission electron microscopy (Black arrow, autophagosome; White arrow, autophagolysosome, scale bar:2 μm, 0.5 μm). **(G–H)** Representative images of immunofluorescence and semi-quantitative analyses of SIRT1 fluorescence intensity in NP cells, imaged by fluorescence microscopy (scale bar = 50 μm). **p < 0.01, ***p < 0.001. Data presented as means ± SD.

To assess whether PEMF regulates NP cell autophagy, we examined LC3B and p62/SQSTM1 levels in NP cells, using western blot. As depicted in Figures 3A–D, after PEMF treatment, the ratio of LC3B-II/LC3B-I increased significantly, whereas, p62 levels dropped significantly. Notably, SIRT1 levels were markedly up-regulated, which is in accordance with autophagic activation. In addition, we evaluated autophagosomes and autophagolysosomes, two key structures associated with autophagy, using TEM. Compared to control cells, the PEMF-treated NP cells displayed more cytoplasmic autophagosomes and autophagolysosomes, thereby confirming autophagic activation in PEMF-treated NP cells (Figures 3E,F).

### PEMF Regulates ECM Related Makers Expression *via* Autophagy

**FIGURE 4:**
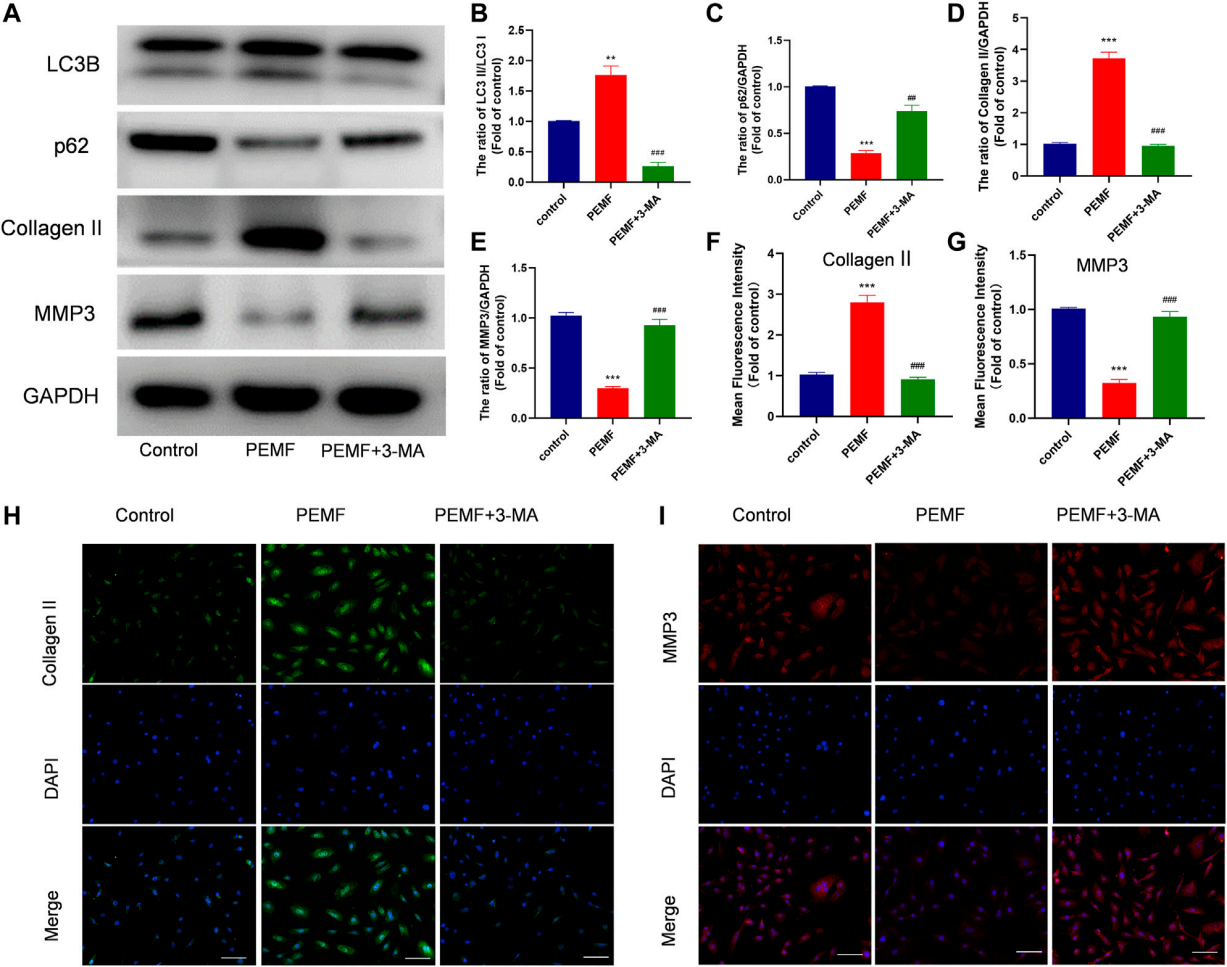
PEMF regulates ECM related makers expression *via* autophagy **(A–E)** Western blot and semi-quantitative analyses of Collagen II, MMP-3, p62, and LC3B protein levels (n = 3). **(F–I)** Representative images of immunofluorescence and semi-quantitative analyses of Collagen II and MMP-3 fluorescence intensity in NP cells, imaged by fluorescence microscopy (scale bar = 50 μm). **p < 0.01 vs. control, ***p < 0.001 vs. control, ##*p* < 0.01 vs. PEMF, ###*p* < 0.001 vs. PEMF. Data presented as means ± SD.

Next, we examined whether autophagy regulates the PEMF-mediated regulation of ECM related makers expression in NP cells. As illustrated in Figure 4A, our western blot data revealed that PEMF treatment markedly reduced MMP3 levels, while increasing type 2 collagen levels, and this effect was reversed by 3-MA exposure (Figures 4B–E). In addition, we used immunofluorescence to assess type 2 collagen and MMP3 expressions in PEMF treated and un-treated NP cells. Based on our results, type 2 collagen expression increased remarkably, while MMP3 expression decreased after PEMF treatment. Conversely, the autophagy inhibitor 3-MA reversed this phenomenon (Figures 4F–I). Based on these evidences, PEMF regulates degenerative makers expression by inducing autophagic activation.

### PEMF Regulates ECM Related Makers Expression *via* SIRT1-Stimulated Autophagic Activation

**FIGURE 5:**
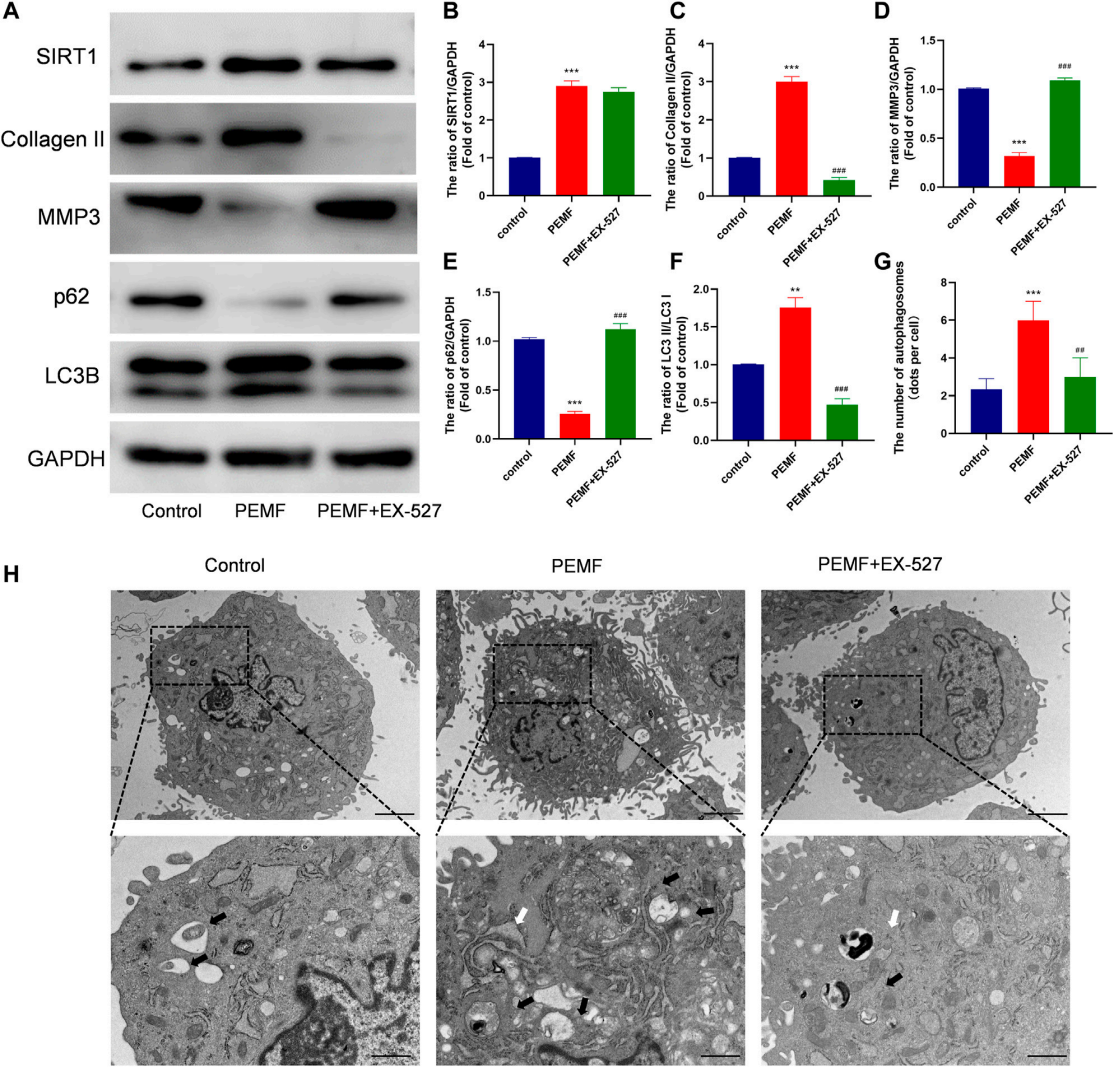
PEMF regulates ECM related makers expression *via* SIRT1-stimulated autophagic activation **(A–F)** Western blot and semi-quantitative analyses of SIRT1, Collagen II, MMP-3, p62 and LC3B protein levels (n = 3). **(G–H)** Autophagosomes and autophagolysosomes, as visualized by transmission electron microscopy (Black arrow, autophagosome; White arrow, autophagolysosome, scale bar:2μm, 0.5 μm). **p < 0.01 vs. control, ***p < 0.001 vs. control, ##*p* < 0.01 vs. PEMF, ###*p* < 0.001 vs. PEMF. Data expressed as means ± SD.

It is well known that Sirt1 is an upstream regulator of autophagy. Interestingly, we found that PEMF treatment markedly increased Sirt1 expression (Figures 3G,H). We, next, examined the relationship between Sirt1, autophagy, and ECM related genes expression in NP cells. We employed EX-527 as a Sirt1 enzyme activity inhibitor, and revealed that EX-527 had no effect on Sirt1 expression. Based on our results, EX-527 treatment inhibited PEMF-induced autophagy, as evidenced by an increase in the LC3-II/LC3-I ratio and p62 levels in the EX 527 group, compared to the PEMF group (Figures 5A–F). Our TEM results revealed that the number of autophagosomes and autophagolysosomes in the EX-527 treated NP cells was also significantly reduced, compared to the PEMF treated NP cells (Figures 5G,H). Taken together, EX-527 inhibited the PEMF-mediated induction of ECM synthesis in NP cells, as evidenced by the marked decrease in collagen II expression and the simultaneous increase in MMP3 expression. In all, our results suggest that PEMF promotes NP cell ECM synthesis by stimulating the Sirt1-mediated autophagy pathway.

### PEMF Improved Rat IVD Degeneration in a Long-Term Treatment

**FIGURE 6:**
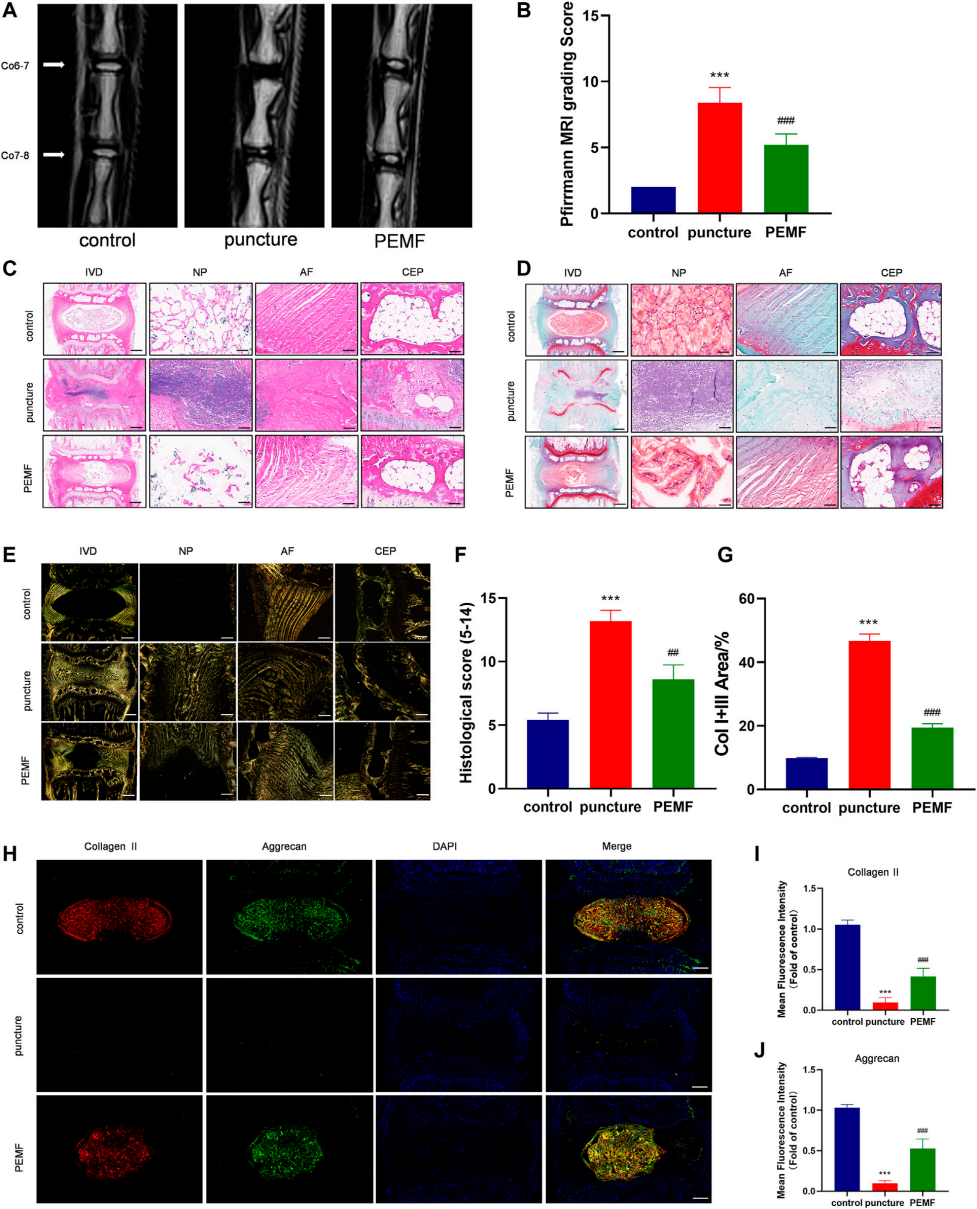
PEMF exposure improved rat IVD degeneration *in vivo*. **(A–B)** Representative images of IVD tissue MRI and Pffirrmann score after 8 weeks of PEMF exposure. **(C–E)** Representative images of IVD tissue HE, S-O staining, and Polarized Sirius Red image (level: Co6-7; sagittal; scale bar: 500 µm, 100 µm). **(F–G)** Corresponding histological score and percentage of type I and type III collagen area. **(H–J)** Representative images of immunofluorescence and semi-quantitative analyses of Collagen II and Aggrecan fluorescence intensity in IVD tissues, imaged by fluorescence microscopy (sagittal; scale bar = 500 μm). ***p < 0.001 vs. control, ##*p* < 0.01 vs. puncture, ###*p* < 0.001 vs. puncture. Data expressed as means ± SD.

To examine the PEMF-mediated regulation of IVD degeneration *in vivo*, we established an IVD degeneration model by puncturing the IVD of rat tail. Firstly, we measured the changes in the weight of the rats to monitor whether PEMF would affect other tissues in rats. No significance change was observed in the weight of rats in each group at 2w, 4w and 8w timepoint with or without PEMF stimulation, which indicates that PEMF is safe and has no side effects in rats (Supplementary Figure 2A). We next assessed IVD degeneration *via* MRI after 8 weeks. Based on our results, the T2-weighted signal intensity of the PEMF rats was stronger, compared to the puncture only rats (Figure 6A). Moreover, the Pfirrmann score grading of IVD degeneration showed that the PEMF treatment group had markedly reduced scores, relative to the puncture-only rats (Figure 6B). Furthermore, using H&E and Safranin-O staining (Figures 6C,D), we revealed that the number of NP tissues in the PEMF treatment group was greater, relative to the puncture-only group, and the integrity of AF and CEP was also significantly better than the puncture-only group. In terms of the histological score, the PEMF-treated rats had markedly reduced scores, compared to the puncture rats (Figure 6F). Additionally, based on the Sirius red staining, the amount of collagen I and collagen III in the PEMF-treated rats were markedly reduced, relative to the puncture rats (Figures 6E,G). Collectively, the above data indicate that PEMF application attenuates the process of IVD degeneration *in vivo*.

### PEMF Induces SIRT1-Mediated Autophagy *in vivo*

**FIGURE 7:**
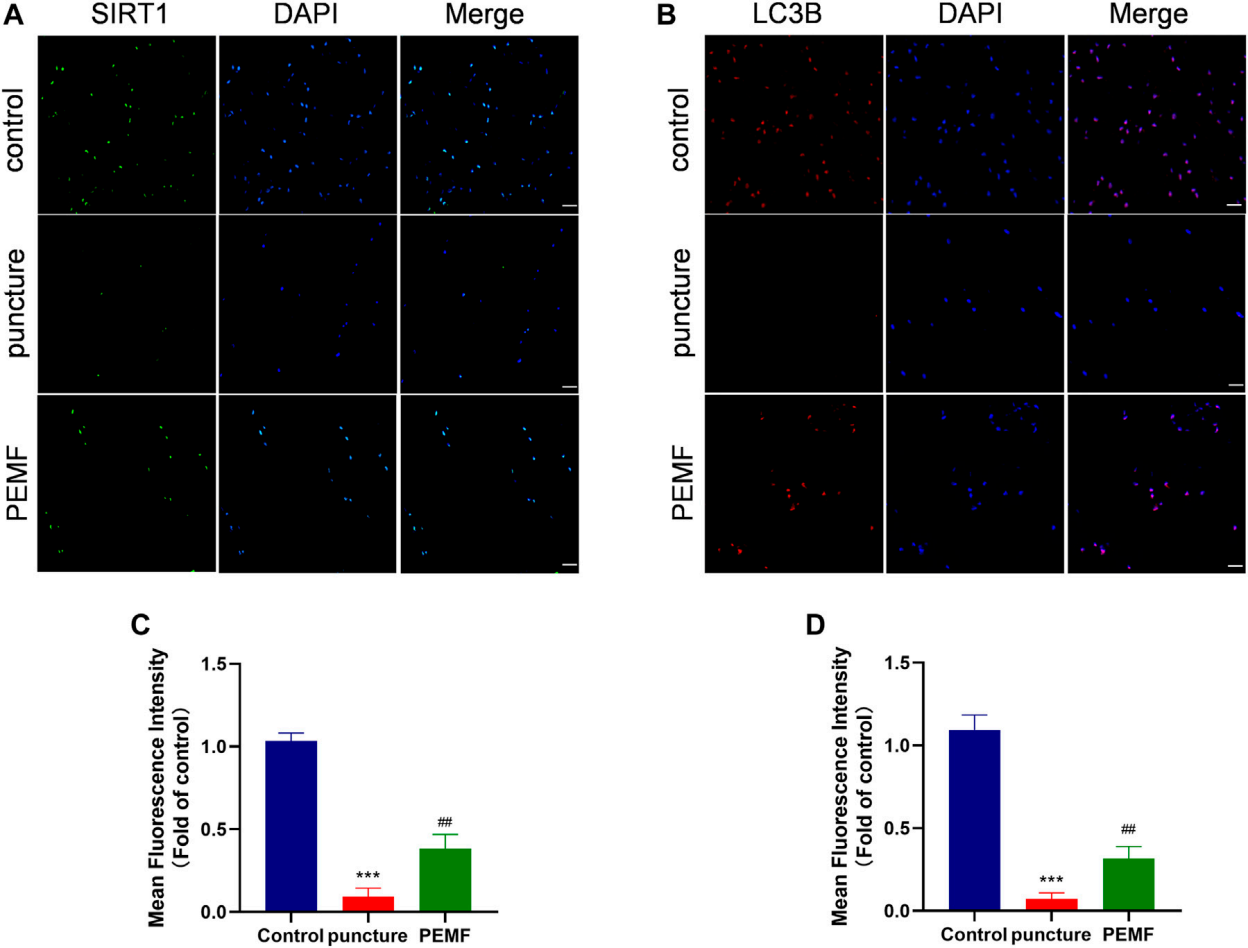
**(A–D)** Representative images of immunofluorescence and semi-quantitative analyses of SIRT1 and LC3B fluorescence intensity in IVD tissues, imaged by fluorescence microscopy (scale bar = 50 μm). ***p < 0.001 vs. control, ##*p* < 0.01 vs. puncture. Data expressed as means ± SD.

We next examined whether PEMF induces the SIRT1-autophagy network *in vivo*. Relative to the puncture only group, immunofluorescence staining revealed that LC3B and SIRT1-positive cells were remarkably enhanced in the PEMF treated rats (Figures 7A,B). In addition, based on immunofluorescence staining, Aggrecan and collagen II expressions were also markedly elevated in the PEMF treated rats, relative to the puncture only rats (Figures 6H–J). Taken together, our *in vivo* investigation confirmed that PEMF preserve healthy ECM, and limit both collagen II and aggrecan degeneration in NP cells *via* the SIRT1-autophagic network.

## Discussion

Herein, we employed the human degenerated NP cells and a rat IVD puncture model to systematically assess the therapeutic outcome of PEMF on IVD degeneration. In our *in vitro* investigation, we revealed that PEMF indeed enhances ECM synthesis *via* the SIRT1-dependent autophagic network. We also assessed PEMF therapeutic efficacy in a rat IVD degeneration model. Based on our research, PEMF indeed delays the progression of IVD degeneration and we confirmed that the PEMF-mediated therapeutic effect on IVD degeneration involves the SIRT1-autophagic network.

Prior investigations revealed that alterations in the ECM metabolism in IVD tissue disrupt ECM structure, thereby reducing IVD tolerance to mechanical loads (Novais et al., 2021a; Hu S. et al., 2021; Rajasekaran et al., 2021). Generally, reduced ECM production and elevated ECM degradation are characteristic of IVD degeneration. Therefore, developing a treatment method that promotes ECM repair of IVD tissue is essential to treating IVD degeneration.

It is well known that PEMF exerts its therapeutic effect *via* regulation of metabolic pathways (Cadossi et al., 2020). This occurs *via* regulation of cell proliferation, differentiation, and maturation, particularly in the treatment of musculoskeletal diseases (Yang et al., 2021; Liu et al., 2022). PEMF was reported to improve treatment of various bone diseases, such as, cartilage defects, tendon repair, osteoarthritis, osteoporosis, and so on (Reilingh et al., 2016; Wang et al., 2019; Dolkart et al., 2021). In recent years, clinical studies have shown that PEMF also has a significant relieving effect on the pain caused by IVD degeneration (Foley-Nolan et al., 1990; Sutbeyaz et al., 2006; Alvarez et al., 2019). In a placebo-controlled study evaluating the efficacy of PEMF treatment in patients with chronic neck pain, patients who received PEMF showed significant improvements in neck pain and range of motion compared to a control group (Foley-Nolan et al., 1990). In another clinical study evaluating the efficacy of PEMF for pain in patients with cervical disc herniation, patients showed significant improvement in pain scores from pre-treatment to week 12 after treatment compared to control group (Alvarez et al., 2019). In basic science research, since PEMFs can generate oscillating magnetic fields, they are able to affect and alter cellular functions by inducing vibrations of free ions on the cell membrane surface (Ganesan et al., 2009). It has also been suggested that PEMFs can modulate downstream signal transduction pathways and cell surface receptor expression/activation to modulate cellular functions such as proliferation, differentiation, extracellular matrix (ECM) synthesis, and intercellular communication (Delle Monache et al., 2013; Viganò et al., 2016; Furuya-Kanamori et al., 2020). In IVD cells, previous studies demonstrated that PEMF induced upregulation of matrix synthesis through bone morphogenetic protein (BMP) signaling (Schmidt-Rohlfing et al., 2008; Okada et al., 2013). Therefore, in this study, we examined the influence of PEMF on degenerated NP cells and IVD degeneration animal models. In *in vitro* experiments, we demonstrated that PEMF promotes expression of collagen II (related to ECM synthesis), while reducing MMP3 (related to ECM catabolism) levels. However, no significance change was observed in cell characterizations, including cell apoptosis, viability and cell morphology. Even though the positive effect of PEMF in degenerative IVD cells was demonstrated, the role of PEMF in non-degenerated cells remains to be studied. In *in vivo* studies, we successfully constructed a rat tail IVD degeneration model. Then, we applied PEMF systematically to the rats. To better understand the systemic effects of PEMF on rats, we measured body weight of rats at 2w, 4w and 8w timepoint, and no significance change was observed in each group, which confirmed that PEMF might be a safe noninvasive therapy. It has been shown that PEMF modulates biological functions of many tissues under pathological conditions, including injuries in bones, tendons, muscles and nervous system (Gessi et al., 2019; Huegel et al., 2020; Dolkart et al., 2021). In this study we mainly focus on the PEMF effect on disc degeneration induced by tail puncture. Our results suggested that PEMF alleviates IVD degeneration. Moreover, radiographic and histological results showed that no significant change was observed in the tissue surrounding the disc, which indicates that PEMF might be a safe and effective treatment in the future clinical use. Despite some relatively studies of PEMF in IVD degeneration, little is known about the underlying mechanism of such therapies.

Autophagy is an evolutionarily conserved modulatory behavior that sustains cell homeostasis *via* lysosomal-dependent degradation of nonfunctioning organelles and misfolded proteins (Kitada and Koya 2021). Previous studies reported that autophagy is inhibited in degenerative diseases, including degeneration of IVD (Kim et al., 2021; Valenti et al., 2021). Therefore, autophagy activation can play a protective role. For example, melatonin alleviates IVD degeneration by promoting autophagy (Chen C. G. et al., 2020). Another Studies have shown that activation of autophagy in chondrocytes inhibits MMP activity and exerts a protective effect on chondrocytes, thereby blocking cartilage degeneration (Takayama et al., 2014; Vasheghani et al., 2015). In order to examine whether PEMF plays a protective role by promoting ECM synthesis *via* autophagy in IVD degeneration, we employed the classical autophagy inhibitor 3-MA. According to our analysis, 3-MA treatment markedly inhibited PEMF-mediated autophagy. This was achieved *via* a significant decrease in p62 protein levels and a simultaneous increase in the LC3-II/LC3-I ratio. Conversely, PEMF accelerated ECM synthesis by elevating collagen II levels, and inhibiting the catabolic process (i.e., reducing MMP3 levels). Moreover, these effects were reversed by 3-MA administration. Recently, studies have shown that various matrix molecules, including glycosaminoglycans, collagen, constituting the intervertebral disc and cartilage ECM, regulate autophagy through mTOR signaling (Chen F. et al., 2020; Zhang et al., 2021). Moreover, autophagy can in turn regulate ECM composition, directly or indirectly altering cellular function, i.e., the secretion of ECM molecules (Novais et al., 2021b). Given the complexity of crosstalk between autophagy and ECM, further insights into the molecular effects of crosstalk between ECM and autophagy during disc degeneration will be critical in the future research.

In our earlier work, we demonstrated a negative correlation between SIRT1 expression and IVD degeneration (Wang et al., 2020). In order to elucidate potential associations between SIRT1, autophagy, and IVD degeneration, we employed a classic SIRT1 enzyme activity suppressor EX-527 in our *in vitro* studies. Based on results, EX-527 has no effect on SIRT1 expression, but can severely reduce its enzymatic activity. We demonstrated that EX-527 effectively inhibits PEMF-induced autophagic activation, along with collagen II synthesis and MMP3 decomposition. Using an IVD rat model, we also validated that PEMF successfully improves puncture-stimulated IVD degeneration by elevating SIRT1 levels and activating autophagy. Although previous reports hold varying opinions on PEMF and SIRT1 regulation (Jeong et al., 2017; Patruno et al., 2020; He et al., 2021), our research confirmed that PEMF indeed promotes the expression of SIRT1 and autophagy, and delays the process of IVD degeneration.

## Conclusion

In conclusion, our research provides evidence that PEMF promotes the SIRT1-autophagic network and reduces IVD degeneration in cells and animal models. These results provide new insights of PEMF in treating IVD degeneration and highlight their therapeutic potential in IVD degeneration. Hence, it is our hope that the present study will promote future application of PEMF as a potential therapeutic treatment for preventing IVD degeneration in clinical use.

## Data Availability

The original contributions presented in the study are included in the article/Supplementary Material, further inquiries can be directed to the corresponding authors.

## References

[B1] Alvarez L. X. McCue J. Lam N. K. Askin G. Fox P. R. (2019). Effect of Targeted Pulsed Electromagnetic Field Therapy on Canine Postoperative Hemilaminectomy: A Double-Blind, Randomized, Placebo-Controlled Clinical Trial. J. Am. Anim. Hosp. Assoc. 55, 83–91. 10.5326/jaaha-ms-6798 30776260

[B2] Bloise N. Patrucco A. Bruni G. Montagna G. Caringella R. Fassina L. (2020). *In Vitro* Production of Calcified Bone Matrix onto Wool Keratin Scaffolds via Osteogenic Factors and Electromagnetic Stimulus. Materials (Basel) 13, 3052. 10.3390/ma13143052 PMC7411850 32650489

[B3] Cadossi R. Massari L. Racine-Avila J. Aaron R. K. (2020). Pulsed Electromagnetic Field Stimulation of Bone Healing and Joint Preservation: Cellular Mechanisms of Skeletal Response. J. Am. Acad. Orthop. Surg. Glob. Res. Rev. 4, e1900155. 10.5435/JAAOSGlobal-D-19-00155 33970582 PMC7434032

[B4] Caliogna L. Medetti M. Bina V. Brancato A. M. Castelli A. Jannelli E. (2021). Pulsed Electromagnetic Fields in Bone Healing: Molecular Pathways and Clinical Applications. Int. J. Mol. Sci. 22, 7403. 10.3390/ijms22147403 34299021 PMC8303968

[B5] Cashin A. G. Folly T. Bagg M. K. Wewege M. A. Jones M. D. Ferraro M. C. (2021). Efficacy, Acceptability, and Safety of Muscle Relaxants for Adults with Non-specific Low Back Pain: Systematic Review and Meta-Analysis. Bmj 374, n1446. 10.1136/bmj.n1446 34233900 PMC8262447

[B6] Chan A. K. Tang X. Mummaneni N. V. Coughlin D. Liebenberg E. Ouyang A. (2019). Pulsed Electromagnetic fields Reduce Acute Inflammation in the Injured Rat-Tail Intervertebral Disc. JOR Spine 2, e1069. 10.1002/jsp2.1069 31891118 PMC6920683

[B7] Chen C. G. Gubbiotti M. A. Kapoor A. Han X. Yu Y. Linhardt R. J. (2020a). Autophagic Degradation of HAS2 in Endothelial Cells: A Novel Mechanism to Regulate Angiogenesis. Matrix Biol. 90, 1–19. 10.1016/j.matbio.2020.02.001 32084457

[B8] Chen F. Liu H. Wang X. Li Z. Zhang J. Pei Y. (2020b). Melatonin Activates Autophagy via the NF-Κb Signaling Pathway to Prevent Extracellular Matrix Degeneration in Intervertebral Disc. Osteoarthritis and Cartilage 28, 1121–1132. 10.1016/j.joca.2020.05.011 32470597

[B9] Cieza A. Causey K. Kamenov K. Hanson S. W. Chatterji S. Vos T. (2021). Global Estimates of the Need for Rehabilitation Based on the Global Burden of Disease Study 2019: a Systematic Analysis for the Global Burden of Disease Study 2019. Lancet 396, 2006–2017. 10.1016/S0140-6736(20)32340-0 33275908 PMC7811204

[B10] Dai F. Yu P. Yu Z. Jiang H. Ma Z. Liu J. (2021). Yiqi Huoxue Recipe Delayed Intervertebral Disc Degeneration by Activating Autophagy. Front. Pharmacol. 12, 705747. 10.3389/fphar.2021.705747 34483910 PMC8416448

[B11] Delle Monache S. Angelucci A. Sanità P. Iorio R. Bennato F. Mancini F. (2013). Inhibition of Angiogenesis Mediated by Extremely Low-Frequency Magnetic Fields (ELF-MFs). PLoS One 8, e79309. 10.1371/journal.pone.0079309 24244477 PMC3828379

[B12] Dolkart O. Kazum E. Rosenthal Y. Sher O. Morag G. Yakobson E. (2021). Effects of Focused Continuous Pulsed Electromagnetic Field Therapy on Early Tendon-To-Bone Healing. Bone Jt. Res. 10, 298–306. 10.1302/2046-3758.105.bjr-2020-0253.r2 PMC8160030 33934605

[B13] Foley-Nolan D. Barry C. Coughlan R. J. O'Connor P. Roden D. (1990). Pulsed High Frequency (27MHz) Electromagnetic Therapy for Persistent Neck Pain: A Double Blind, Placebo-Controlled Study of 20 Patients. Orthopedics 13, 445–451. 10.3928/0147-7447-19900401-10 2185460

[B14] Foster N. E. Anema J. R. Cherkin D. Chou R. Cohen S. P. Gross D. P. (2018). Prevention and Treatment of Low Back Pain: Evidence, Challenges, and Promising Directions. The Lancet 391, 2368–2383. 10.1016/s0140-6736(18)30489-6 29573872

[B15] Francisco V. Pino J. González-Gay M. Á. Lago F. Karppinen J. Tervonen O. (2022). A New Immunometabolic Perspective of Intervertebral Disc Degeneration. Nat. Rev. Rheumatol. 18, 47–60. 10.1038/s41584-021-00713-z 34845360

[B16] Furuya-Kanamori L. Xu C. Lin L. Doan T. Chu H. Thalib L. (2020). P Value-Driven Methods Were Underpowered to Detect Publication Bias: Analysis of Cochrane Review Meta-Analyses. J. Clin. Epidemiol. 118, 86–92. 10.1016/j.jclinepi.2019.11.011 31743750

[B17] Ganesan K. Gengadharan A. C. Balachandran C. Manohar B. M. Puvanakrishnan R. (2009). Low Frequency Pulsed Electromagnetic Field-Aa Viable Alternative Therapy for Arthritis. Indian J. Exp. Biol. 47, 939–948. 20329696

[B18] Gessi S. Merighi S. Bencivenni S. Battistello E. Vincenzi F. Setti S. (2019). Pulsed Electromagnetic Field and Relief of Hypoxia-Induced Neuronal Cell Death: The Signaling Pathway. J. Cel Physiol (Epub ahead of print). 10.1002/jcp.28149 30656694

[B19] He G.-L. Wang Z.-Z. Yu X.-T. Shen T.-T. Luo Z. Li P. (2021). The Involvement of Microglial CX3CR1 in Heat Acclimation-Induced Amelioration of Adult Hippocampal Neurogenesis Impairment in EMF-Exposed Mice. Brain Res. Bull. 177, 181–193. 10.1016/j.brainresbull.2021.09.018 34555433

[B20] Hu J. Yan Q. Jiang H. Xu C. Chen Y. Yuan W. (2021a). A Decrease in IL-33 Regulates Matrix Degradation and Apoptosis in Intervertebral Disc Degeneration via HIF-1alpha. Am. J. Transl Res. 13, 12724–12733. 34956487 PMC8661207

[B21] Hu S. Chen L. Al Mamun A. Ni L. Gao W. Lin Y. (2021b). The Therapeutic Effect of TBK1 in Intervertebral Disc Degeneration via Coordinating Selective Autophagy and Autophagic Functions. J. Adv. Res. 30, 1–13. 10.1016/j.jare.2020.08.011 34026282 PMC8132185

[B22] Huegel J. Boorman‐Padgett J. F. Nuss C. A. Raja H. A. Chan P. Y. Kuntz A. F. (2020). Effects of Pulsed Electromagnetic Field Therapy on Rat Achilles Tendon Healing. J. Orthop. Res. 38, 70–81. 10.1002/jor.24487 31595543 PMC6917903

[B23] Jeong W.-Y. Kim J.-B. Kim H.-J. Kim C.-W. (2017). Extremely Low-Frequency Electromagnetic Field Promotes Astrocytic Differentiation of Human Bone Marrow Mesenchymal Stem Cells by Modulating SIRT1 Expression. Biosci. Biotechnol. Biochem. 81, 1356–1362. 10.1080/09168451.2017.1308243 28351214

[B24] Jiang D.-P. Li J.-h. Zhang J. Xu S.-L. Kuang F. Lang H.-Y. (2016). Long-term Electromagnetic Pulse Exposure Induces Abeta Deposition and Cognitive Dysfunction through Oxidative Stress and Overexpression of APP and BACE1. Brain Res. 1642, 10–19. 10.1016/j.brainres.2016.02.053 26972535

[B25] Jing D. Shen G. Huang J. Xie K. Cai J. Xu Q. (2010). Circadian Rhythm Affects the Preventive Role of Pulsed Electromagnetic fields on Ovariectomy-Induced Osteoporosis in Rats. Bone 46, 487–495. 10.1016/j.bone.2009.09.021 19782781

[B26] Kavand H. Haghighipour N. Zeynali B. Seyedjafari E. Abdemami B. (2016). Extremely Low Frequency Electromagnetic Field in Mesenchymal Stem Cells Gene Regulation: Chondrogenic Markers Evaluation. Artif. Organs 40, 929–937. 10.1111/aor.12696 27086585

[B27] Kim J. W. Jeon N. Shin D. E. Lee S. Y. Kim M. Han D. H. (2021). Regeneration in Spinal Disease: Therapeutic Role of Hypoxia-Inducible Factor-1 Alpha in Regeneration of Degenerative Intervertebral Disc. Int. J. Mol. Sci. 22, 5281. 10.3390/ijms22105281 34067899 PMC8155933

[B28] Kitada M. Koya D. (2021). Autophagy in Metabolic Disease and Ageing. Nat. Rev. Endocrinol. 17, 647–661. 10.1038/s41574-021-00551-9 34508250

[B29] Knezevic N. N. Candido K. D. Vlaeyen J. W. S. Van Zundert J. Cohen S. P. (2021). Low Back Pain. Lancet 398, 78–92. 10.1016/S0140-6736(21)00733-9 34115979

[B30] Kritschil R. Scott M. Sowa G. Vo N. (2021). Role of Autophagy in Intervertebral Disc Degeneration. J. Cel Physiol 237, 1266. 10.1002/jcp.30631 PMC8866220 34787318

[B31] Li Z. Liu T. Feng Y. Tong Y. Jia Y. Wang C. (2022). PPARγ Alleviates Sepsis-Induced Liver Injury by Inhibiting Hepatocyte Pyroptosis via Inhibition of the ROS/TXNIP/NLRP3 Signaling Pathway. Oxidative Med. Cell Longevity 2022, 1–15. 10.1155/2022/1269747 PMC8818407 35136484

[B32] Liu L. Huang K. Li W. Qiu R. Fang Y. Huang Y. (2021). Molecular Imaging of Collagen Destruction of the Spine. ACS Nano 15, 19138–19149. 10.1021/acsnano.1c07112 34738460

[B33] Mei L. Zheng Y. Ma T. Xia B. Gao X. Hao Y. (2021). (-)-Epigallocatechin-3-gallate Ameliorates Intervertebral Disc Degeneration through Reprogramming of the Circadian Clock. Front. Pharmacol. 12, 753548. 10.3389/fphar.2021.753548 34803694 PMC8599576

[B34] Miller S. L. Coughlin D. G. Waldorff E. I. Ryaby J. T. Lotz J. C. (2016). Pulsed Electromagnetic Field (PEMF) Treatment Reduces Expression of Genes Associated with Disc Degeneration in Human Intervertebral Disc Cells. Spine J. 16, 770–776. 10.1016/j.spinee.2016.01.003 26780754

[B35] Novais E. J. Choi H. Madhu V. Suyama K. Anjo S. I. Manadas B. (2021a). Hypoxia and Hypoxia-Inducible Factor-1α Regulate Endoplasmic Reticulum Stress in Nucleus Pulposus Cells. Am. J. Pathol. 191, 487–502. 10.1016/j.ajpath.2020.11.012 33307037 PMC7927276

[B36] Novais E. J. Tran V. A. Johnston S. N. Darris K. R. Roupas A. J. Sessions G. A. (2021b). Long-term Treatment with Senolytic Drugs Dasatinib and Quercetin Ameliorates Age-dependent Intervertebral Disc Degeneration in Mice. Nat. Commun. 12, 5213. 10.1038/s41467-021-25453-2 34480023 PMC8417260

[B37] Okada M. Kim J. H. Hutton W. C. Yoon S. T. (2013). Upregulation of Intervertebral Disc-Cell Matrix Synthesis by Pulsed Electromagnetic Field Is Mediated by Bone Morphogenetic Proteins. J. Spinal Disord. Tech. 26, 167–173. 10.1097/bsd.0b013e31823d36cf 22105104

[B38] Parate D. Franco-Obregón A. Fröhlich J. Beyer C. Abbas A. A. Kamarul T. (2017). Enhancement of Mesenchymal Stem Cell Chondrogenesis with Short-Term Low Intensity Pulsed Electromagnetic fields. Sci. Rep. 7, 9421. 10.1038/s41598-017-09892-w 28842627 PMC5572790

[B39] Patruno A. Costantini E. Ferrone A. Pesce M. Diomede F. Trubiani O. (2020). Short ELF-EMF Exposure Targets SIRT1/Nrf2/HO-1 Signaling in THP-1 Cells. Int. J. Mol. Sci. 21, 7284. 10.3390/ijms21197284 PMC7582394 33023074

[B40] Rajasekaran S. Thangavel C. Djuric N. Raveendran M. Soundararajan D. C. R. Nayagam S. M. (2021). Profiling Extra Cellular Matrix Associated Proteome of Human Fetal Nucleus Pulposus in Search for Regenerative Targets. Sci. Rep. 11, 19013. 10.1038/s41598-021-97620-w 34561485 PMC8463528

[B41] Reilingh M. L. van Bergen C. J. A. Gerards R. M. van Eekeren I. C. de Haan R. J. Sierevelt I. N. (2016). Effects of Pulsed Electromagnetic Fields on Return to Sports after Arthroscopic Debridement and Microfracture of Osteochondral Talar Defects. Am. J. Sports Med. 44, 1292–1300. 10.1177/0363546515626544 26903214

[B42] Schmidt-Rohlfing B. Silny J. Woodruff S. Gavenis K. (2008). Effects of Pulsed and Sinusoid Electromagnetic fields on Human Chondrocytes Cultivated in a Collagen Matrix. Rheumatol. Int. 28, 971–977. 10.1007/s00296-008-0565-0 18389240

[B43] Sobajima S. Kompel J. F. Kim J. S. Wallach C. J. Robertson D. D. Vogt M. T. (2005). A Slowly Progressive and Reproducible Animal Model of Intervertebral Disc Degeneration Characterized by MRI, X-Ray, and Histology. Spine 30, 15–24. 10.1097/01.brs.0000148048.15348.9b 15626975

[B44] Sun Z. Liu B. Liu Z.-H. Song W. Wang D. Chen B.-Y. (2020). Notochordal-Cell-Derived Exosomes Induced by Compressive Load Inhibit Angiogenesis via the miR-140-5p/Wnt/β-Catenin Axis. Mol. Ther. - Nucleic Acids 22, 1092–1106. 10.1016/j.omtn.2020.10.021 33294295 PMC7691158

[B45] Sutbeyaz S. T. Sezer N. Koseoglu B. F. (2006). The Effect of Pulsed Electromagnetic fields in the Treatment of Cervical Osteoarthritis: a Randomized, Double-Blind, Sham-Controlled Trial. Rheumatol. Int. 26, 320–324. 10.1007/s00296-005-0600-3 15986086

[B46] Takayama K. Kawakami Y. Kobayashi M. Greco N. Cummins J. H. Matsushita T. (2014). Local Intra-articular Injection of Rapamycin Delays Articular Cartilage Degeneration in a Murine Model of Osteoarthritis. Arthritis Res. Ther. 16, 482. 10.1186/s13075-014-0482-4 25403236 PMC4269094

[B47] Tu J. Li W. Yang S. Yang P. Yan Q. Wang S. (2021). Single-Cell Transcriptome Profiling Reveals Multicellular Ecosystem of Nucleus Pulposus during Degeneration Progression. Adv. Sci. (Weinh) 9, e2103631. 10.1002/advs.202103631 34825784 PMC8787427

[B48] Valenti M. T. Dalle Carbonare L. Zipeto D. Mottes M. (2021). Control of the Autophagy Pathway in Osteoarthritis: Key Regulators, Therapeutic Targets and Therapeutic Strategies. Int. J. Mol. Sci. 22, 2700. 10.3390/ijms22052700 33800062 PMC7962119

[B49] Varani K. Vincenzi F. Pasquini S. Blo I. Salati S. Cadossi M. (2021). Pulsed Electromagnetic Field Stimulation in Osteogenesis and Chondrogenesis: Signaling Pathways and Therapeutic Implications. Int. J. Mol. Sci. 22, 809. 10.3390/ijms22020809 PMC7830993 33467447

[B50] Vasheghani F. Zhang Y. Li Y.-H. Blati M. Fahmi H. Lussier B. (2015). PPARγ Deficiency Results in Severe, Accelerated Osteoarthritis Associated with Aberrant mTOR Signalling in the Articular Cartilage. Ann. Rheum. Dis. 74, 569–578. 10.1136/annrheumdis-2014-205743 25573665 PMC4345902

[B51] Viganò M. Sansone V. d’Agostino M. C. Romeo P. Perucca Orfei C. de Girolamo L. (2016). Mesenchymal Stem Cells as Therapeutic Target of Biophysical Stimulation for the Treatment of Musculoskeletal Disorders. J. Orthop. Surg. Res. 11, 163. 10.1186/s13018-016-0496-5 27986082 PMC5162101

[B52] Wang D. He X. Wang D. Peng P. Xu X. Gao B. (2020). Quercetin Suppresses Apoptosis and Attenuates Intervertebral Disc Degeneration via the SIRT1-Autophagy Pathway. Front. Cel Dev. Biol. 8, 613006. 10.3389/fcell.2020.613006 PMC7758489 33363176

[B53] Wang T. Yang L. Jiang J. Liu Y. Fan Z. Zhong C. (2019). Pulsed Electromagnetic fields: Promising Treatment for Osteoporosis. Osteoporos. Int. 30, 267–276. 10.1007/s00198-018-04822-6 30603841

[B54] Yang X. Guo H. Ye W. Yang L. He C. (2021). Pulsed Electromagnetic Field Attenuates Osteoarthritis Progression in a Murine Destabilization-Induced Model through Inhibition of TNF-α and IL-6 Signaling. Cartilage 13, 1665s–1675S. 10.1177/19476035211049561 34612715 PMC8804761

[B55] Zhang T. W. Li Z. F. Ding W. Wang H. R. Ding S. L. Han G. J. (2021). Decorin Inhibits Nucleus Pulposus Apoptosis by Matrix‐induced Autophagy via the mTOR Pathway. J. Orthop. Res. 39, 1777–1788. 10.1002/jor.24882 33034924

[B56] Zhi Z. Na T. Jue W. Zhihe Z. Lijun T. (2016). Effects of Pulsed Ultrasound and Pulsed Electromagnetic Field on the Extracellular Matrix Secretion of Rat Bone Marrow Mesenchymal Stem Cell Pellets in Chondrogenesis. Hua Xi Kou Qiang Yi Xue Za Zhi 34, 291–294. 10.7518/hxkq.2016.03.015 27526456 PMC7030833

[B57] Zhong H. Yang C. Gao Y. Cao P. Tian Y. Shen X. (2021). PERK Signaling Activation Restores Nucleus Pulposus Degeneration by Activating Autophagy under Hypoxia Environment. Osteoarthritis Cartilage 30, 341. 10.1016/j.joca.2021.11.005 34767959

[B58] Zhu S. Ge J. Liu Z. Liu L. Jing D. Ran M. (2017). Circadian Rhythm Influences the Promoting Role of Pulsed Electromagnetic Fields on Sciatic Nerve Regeneration in Rats. Front. Neurol. 8, 101. 10.3389/fneur.2017.00101 28360885 PMC5350136

[B59] Zielinski J. Ducray A. D. Moeller A. M. Murbach M. Kuster N. Mevissen M. (2020). Effects of Pulse-Modulated Radiofrequency Magnetic Field (RF-EMF) Exposure on Apoptosis, Autophagy, Oxidative Stress and Electron Chain Transport Function in Human Neuroblastoma and Murine Microglial Cells. Toxicol. Vitro 68, 104963. 10.1016/j.tiv.2020.104963 32777439

