# Supplementary material for: Pulsed Electromagnetic Field Alleviates Intervertebral Disc Degeneration by Activating Sirt1-Autophagy Signaling Network
Source: Front Bioeng Biotechnol. 2022 Mar 21;10:853872. doi: 10.3389/fbioe.2022.853872 (PMC8978825; doi:10.3389/fbioe.2022.853872)
Supplement: Supplementary file 1 [file DataSheet1.docx]

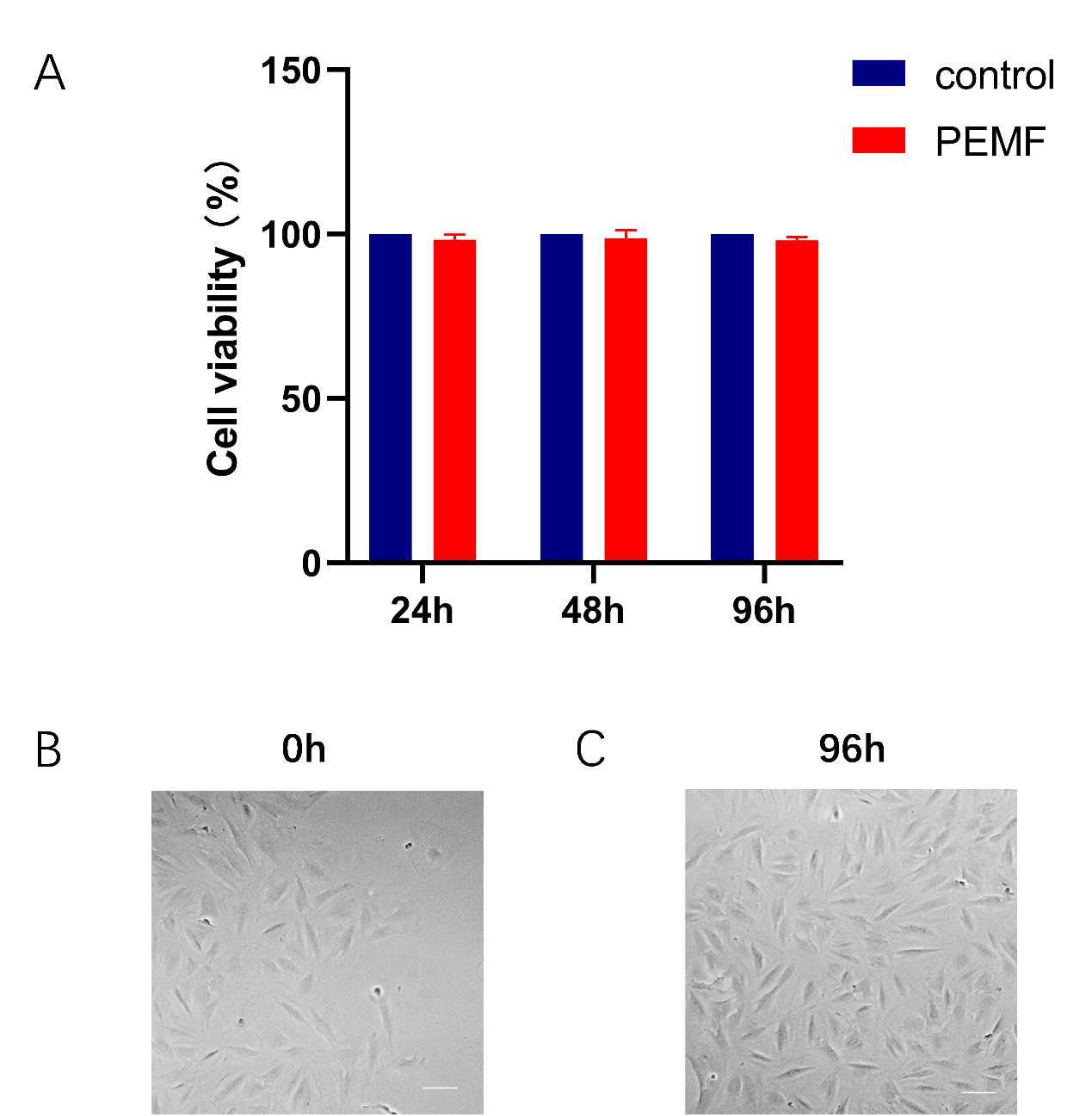


Supplementary Figure 1. (A) CCK-8 results of NP cells viability at time points 24h, 48h, and 96h after 4h/day of PEMF administration. (B-C) NP cells imaged by phase-contrast microscopy with and without PEMF stimulation (scale bar: 50 μm).


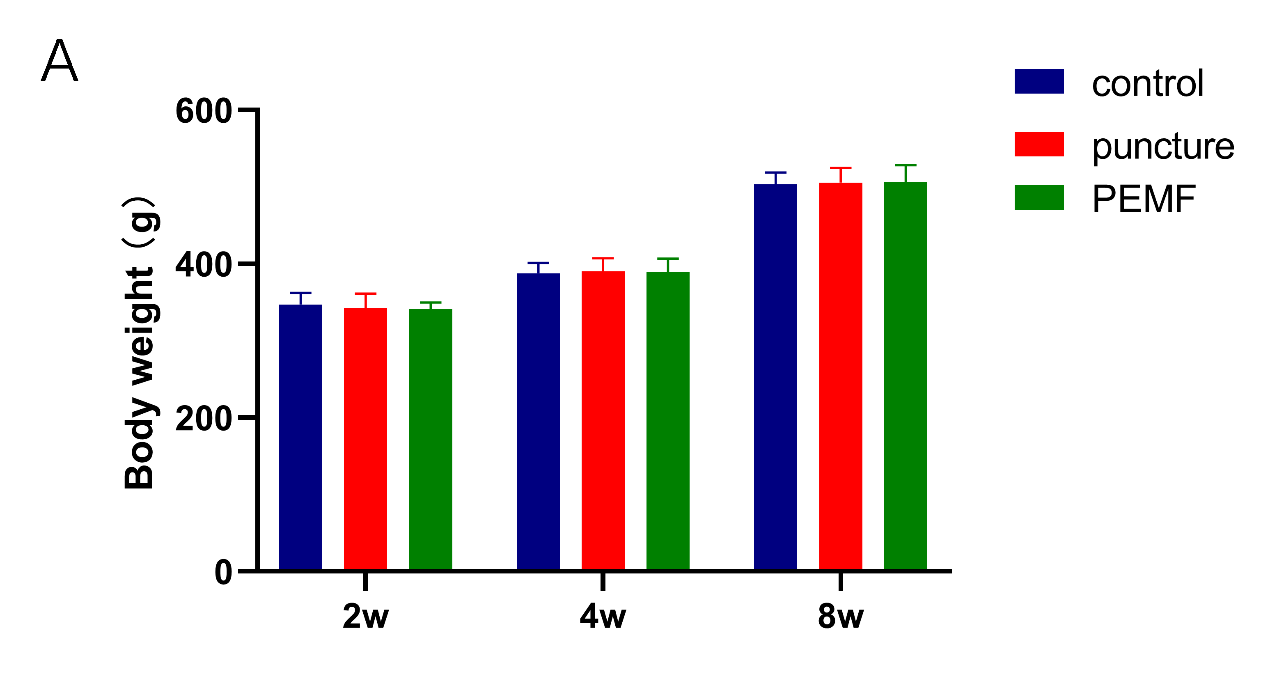


Supplementary Figure 2. (A) Body weight of rats in each group at 2w, 4w and 8w timepoint.
